# Usability Testing of the BRANCH Smartphone App Designed to Reduce Harmful Drinking in Young Adults

**DOI:** 10.2196/mhealth.7836

**Published:** 2017-08-08

**Authors:** Joanna Milward, Paolo Deluca, Colin Drummond, Rod Watson, Jacklyn Dunne, Andreas Kimergård

**Affiliations:** ^1^ Addictions Department King's College London London United Kingdom; ^2^ Health Innovation Network London United Kingdom

**Keywords:** alcohol, drinking, young adults, mHealth, brief intervention, apps, usability testing, user experience, focus group

## Abstract

**Background:**

Electronic screening and brief intervention (eSBI) apps demonstrate potential to reduce harmful drinking. However, low user engagement rates with eSBI reduce overall effectiveness of interventions. As “Digital Natives,” young adults have high expectations of app quality. Ensuring that the design, content, and functionality of an eSBI app are acceptable to young adults is an integral stage to the development process.

**Objective:**

The objective of this study was to identify usability barriers and enablers for an app, BRANCH, targeting harmful drinking in young adults.

**Methods:**

The BRANCH app contains a drinking diary, alcohol reduction goal setting functions, normative drinking feedback, and information on risks and advice for cutting down. The app includes a social feature personalized to motivate cutting down and to promote engagement with a point-based system for usage. Three focus groups were conducted with 20 users who had tested the app for 1 week. A detailed thematic analysis was undertaken.

**Results:**

The first theme, “Functionality” referred to how users wanted an easy-to-use interface, with minimum required user-input. Poor functionality was considered a major usability barrier. The second theme, “Design” described how an aesthetic with minimum text, clearly distinguishable tabs and buttons and appealing infographics was integral to the level of usability. The final theme, “Content” described how participants wanted all aspects of the app to be automatically personalized to them, as well as providing them with opportunities to personalize the app themselves, with increased options for social connectivity.

**Conclusions:**

There are high demands for apps such as BRANCH that target skilled technology users including young adults. Key areas to optimize eSBI app development that emerged from testing BRANCH with representative users include high-quality functionality, appealing aesthetics, and improved personalization.

## Introduction

Electronic screening and brief intervention (eSBI), delivered through devices such as computers, tablets and smartphones, is an increasingly popular method to deliver alcohol brief interventions [1-3]. Meta-analyses demonstrate eSBI to be effective in reducing alcohol consumption by 1 to 2 drinks per week after 6 months compared to controls [4,5]. However the majority of eSBIs that have been evaluated are Web-based, as opposed to app-based programs. A recent systematic review of mobile interventions for alcohol and substance use reported that while mobile delivery of alcohol interventions is an acceptable and effective communication channel, targeting of interventions to specific populations is required [6]. Another enduring challenge for eSBI development is usability. A recent review of online feedback for existing alcohol apps reported that nonintuitive functionality, software malfunctions, and lack of personalization of content are frequently cited criticisms of existing alcohol apps [3,7]. While many eSBI apps are available, the majority are not evidence-based [2,8]. Indeed eSBI is still in its infancy and should be subjected to multiple stages of rigorous development and testing [9].

The app “BRANCH” targets harmful drinking in young adults (18-30 year olds). This study reports the second stage of development of the BRANCH app that aims to evaluate the usability of the prototype app to improve its functioning, content, and design. Usability testing is a crucial stage in the app design process as it provides end user feedback about what does and what does not work in the program. This is an important step as the effectiveness of an electronic intervention has been shown to be associated with its level of usability [10]. Conversely, poor usability is associated with nonusage [11].

Usability testing is widely used in digital health intervention design, and more recently in the development of digital programs for alcohol harm reduction. A range of methods for usability testing exist including questionnaires [12,13], think-aloud observation, and interview-based techniques [14]. For alcohol field, the interview-based techniques are more widely used. Crane et al [7] conducted usability testing on an app designed to reduce alcohol consumption using think aloud testing and semistructured interviews to determine if the features in the app were acceptable and feasible to users and also determine what could be improved. Similarly, Davies [15] assessed the feasibility and acceptability of a digital alcohol harm reduction tool for adolescents, and Dulin [16] conducted usability testing via questionnaires and interviews of a smartphone intervention for adults. These studies highlighted issues of ease-of-use, clarity of information, and appealing design as integral to improvement of the digital tools.

However, the BRANCH app is different from other alcohol harm reduction apps in that it targets young adults specifically. Young adults' preferences for the usability of apps may differ from other groups who have lower usage rates of apps, such as older population. Indeed, young adults have the highest level of smartphone ownership out of all age groups [17]. Referred to as “digital natives,” many are proficient in technology use having grown up being exposed to computers, smartphones, and the Internet [18] *.* It is therefore critical to optimize usability for apps targeting this age group. Moreover, usability data and experiences may be of use in the development of other apps designed to influence substance use among young adults.

The aim of the current study was to explore experiences of app usability, in terms of content, functionality, and design of the BRANCH app to improve user experience.

## Methods

### Design and Setting

Qualitative interviews were chosen as most appropriate to meet the aim of the current project. While other methods such as questionnaires can provide useful overviews of app functioning, the level of fine-grained detail provided can be limited. Qualitative methods provide additional insights as they encourage participants to think about ways to improve usability and identify unanticipated challenges [19]. Focus groups were chosen as the most appropriate method of data collection instead of 1:1 interviews as they allow for views to be developed and discussed, and also for individual opinions to be expressed. As the aim of the study was to identify specific usability issues, the intention was that participants in the focus groups would remind and prompt each other about specific issues they experienced, hence yielding higher identification of usability issues compared to 1:1 interviewing. Ethical approval was obtained from the University Ethics Committee (ref. number HR14/150453).

### Participants

Young adults, aged 18-30 years, who lived in South London and scored 16+ on the alcohol use disorders identification test (AUDIT), indicating harmful drinking (AUDIT score between 16-19) or probable dependence (AUDIT score ≥20) [20].

### Materials

The app “BRANCH” targeted harmful drinking in young adults. Prototype development was informed by 3 studies: (1) a systematic review of engagement promoting strategies for online substance use interventions, (2) a review of user-reviews of existing alcohol eSBI apps available on iTunes and Google Play stores [3], and (3) focus groups with young adults drinking at harmful levels and residing in South London exploring their preferences for content features and style for an alcohol brief intervention app [3]. The prototype was designed iteratively, using a user-centered design approach (UCD) [21,22]. This involved collaboration between the program developers, research team, and target population. The core functions of BRANCH were based on the FRAMES model (feedback, responsibility, advice, menu of options, empathy, and self-efficacy) of alcohol brief interventions [23], which has been previously adapted for eSBI [24]. The FRAMES model is based upon the principles of motivational interviewing, an established and evidence based method to reduce alcohol harm [25]. The core functions included a drinking diary for recording alcohol consumption (see Multimedia Appendix 1) and a goal setting function where users could set weekly goals based on cost, calories, and alcohol units as well as setting a drink free day (see Multimedia Appendix 2). Users monitored their drinking over time and received feedback on it, both descriptively and graphically. Information on drinking risks and cutting down was available to users (see Multimedia Appendix 2).

In addition to the core components, several strategies to optimize engagement with the app were incorporated. In order to tailor the app to young adults, these strategies were developed in collaboration with a user group recruited from the target population. The two main components young adults requested were social features and tailoring of the app to broader wellbeing issues associated with alcohol use [3]. Consequently, the app included a Twitter-style newsfeed enabling interaction between app users, as well as providing personalized notifications, motivational messaging, and reminders based on goals (see Multimedia Appendix 3). The research team could also upload relevant material for young adults, such as links to Web-based articles, YouTube videos, and photos. There was also a personalization feature in which the app users selected their motivations for cutting down drinking when signing up. These motivations were chosen by the user group and included options such as mental health, sugar intake, appearance, and weight. Personalized feedback and targeted information was delivered to users based on their selection of motivators. For example, if a user selected “fitness” as their primary motivator, they would receive tailored messaging on their newsfeed on this topic as well as feedback on how much exercise would be needed to burn off the alcohol calories they had consumed over the last week. Additionally, users were allocated to a team based on these motivators. Users could compare their progress against other users in their team and were awarded points for engaging with the app (see Multimedia Appendix 3). The app was Web-based, and could therefore be accessed from all devices.

### Recruitment

Participants were recruited through Gumtree, an online classified and social community website. Potential participants were invited to take part in a focus group interview to test the app designed to support young adults reducing their alcohol use. A link was provided to an online screening questionnaire where they completed the AUDIT and provided their contact and basic demographic data. Eligible participants were invited to take part. Participants who attended a focus group interview were compensated £30 for their time.

### Data Collection

The focus group participants were provided with the BRANCH app 1 week before and instructed to use it daily over the course of the week to monitor their drinking. Participants were provided with the following specific tasks to complete: (1) set up the app, (2) fill out a weeks’ worth of drinking in the drinks diary, (3) set a goal and 3 drink free days, and (4) join a team and review team feedback (See Multimedia Appendices 1-).

A topic guide was designed to explore the extent to which the participants found the different features acceptable, in terms of content, features, and design. Participants were asked to give their views on their experience of using the different features in the app, focusing around what did and what did not work well. Written informed consent was obtained before commencement of the focus group. The focus group was facilitated by 3 researchers (JM, JD, and RD). They were introduced to participants as researchers who were developing an app to reduce harmful drinking. JM led the group; JD and RD took notes of key themes.

### Data Analysis

Focus group interviews were recorded and transcribed verbatim by a professional transcription company. All data were coded using NVivo qualitative data analysis software (QSR International Pty Ltd. Version 10, 2012). JM coded the transcripts and JD double-coded. Any discrepancies that arose were discussed until a consensus was reached.

A thematic analysis was undertaken as outlined by Braun and Clarke [26]. A deductive approach was used. Usability issues were coded into categories of (1) App content, (2) Functionality, or (3) Design. Each of these categories was considered in terms of being a barrier or an enabler to use or a suggested improvement. Themes were systematically refined by going back and forth between the data and the coding framework.

## Results

A total of 70 people completed the online screening survey. Of these, 32 (46%) scored 16 or more on the AUDIT, were between 18-30 years of age, and lived in South London.

### Participant Characteristics

A total of 20 participants attended 1 of the 3 focus groups with 6 to 7 participants in each over a 1-month period in August 2016. These numbers in each group allowed for meaningful discussions to take place between participants. Of the 20 participants, 18 were female (90%), 10 (50%) were employed, 1 (5%) was unemployed, and 9 (45%) were students. The mean age was 23 years (SD 3.9). The mean AUDIT score across the participants was 21 (SD 5.7).

### Usability Analyses

#### Functionality

This theme reflected how all the participants wanted simple and fast functionality, with features that would minimize the amount of effort, input, and time required from them. Features that had efficient and automated functionality were praised as enablers to usability. For example, being able to quickly complete functions in the app such as posting a newsfeed message, setting up the app, or adding data to the drinking diary via a guided walkthrough.

However, while participants typically appreciated features of the app that functioned well, some still expressed views that there were scopes for improvements. When discussing their experiences of using the app, several participants reported becoming quickly frustrated when a feature was hard-to-use or took too much time. Some expressed strong views that their time was precious and that they did not want to spend unnecessary time inputting data, such as when entering drinks into the drinking diary:

Generally when I’m having a cocktail I can’t put in a brand so it’s difficult...for example, a Long Island Iced Tea...has five different variants of alcohol in it...

P7; Focus group 3

And if you had a double, like a double gin and tonic, I had to put in a single and then a shot because you can’t put in a double.

P3; Focus group 3

Many participants also wanted push notifications (ie, reminders) on their phone to prompt them to use the app. A few wanted reduced scrolling to find information on the newsfeed and a help feature to facilitate app usage. Functionality requests were typically related to increasing the level of “automation” in the app, subsequently reducing the amount of time required to use it. For the majority of the participants, increasing automation seemed to improve usability and make the app more valued.

Another important finding that arose was related to the impact that the quality of the functionality of an app can have on a user’s intentions to interact with it. There was a consensus for deleting apps that did not immediately function as expected:

The NHS Change for Life? Was it yellow?

P1; Focus group 3

Yes, it was that and I hated it. The notifications were awful, it just wouldn’t let me do anything so I was like what’s the point of you? Just delete.

P7; Focus group 3

I really hate apps that do that when they send you notifications as well and you still get emails.

P5; Focus group 3

This underlines a difficult balance between too little and too much output, both of which impact usability. As this quotation suggests, it is not a case of providing users with as much content as possible, instead carefully tailoring the content to the requirements of the individual user. Overall, what participants appeared to want from the functionality of BRANCH were features “at their fingertips,” pressing as few buttons as possible, in a fast and seamless interaction. If this was not achieved then there was a risk of losing the user entirely.

#### Design

The design of the app was the usability issue most discussed in all the focus groups. Participants frequently commented on the need for the app to have a well-considered design, with short pieces of clearly presented text and features that were easily distinguishable from one another. An aesthetically displeasing app seemed to be considered to be a major usability barrier. For instance, many participants commented on how the newsfeed was too text heavy, with large blocks of text, not separated by pictures or colors, which made the information difficult to absorb and certain core features not clearly distinguishable:

Did anyone get any goal related messaging?

JM; Focus group 1

I got something like ‘you went to a barbeque this weekend’...

P7; Focus group 1

No, it wouldn’t have been that, it would have had a star next to it here on the newsfeed...

P1; Focus group 1

Oh yeah, look, oh dear...didn’t achieve your goals.

P3; Focus group 1

This is a critical issue as participants were not aware that certain integral features aimed at reducing alcohol-related harm even existed, as the buttons and tabs through which they were advertised were not easily discernible from other newsfeed content.

While participants seemed to express the view that too much text and a poor design hindered usability, the use of multimedia in the “information about drinking” section was the preferred style of most of the participants. This section had been especially designed with an infographic style, which aims to present complex information in easy-to-digest, short components in a colorful, succinct presentation with lots of pictures (see Multimedia Appendix 2):

I like that, I think that would be better, that style, on the Newsfeed. Because I think I'd be more inclined to read it with the pictures and bits and bobs. Cause that looks more...like if you clicked on that and then there’s something in different colours and...Yeah, looks really good.

63; Focus group 2

From this extract, it is apparent that the design of the app can impact the level of engagement a user has with it; in this case, motivating the user to read through information provided. Appealing designs seemed to promote usage while those which were difficult to digest or read through discouraged usage.

Another significant finding about the design of the app was the importance of consistency in style. While efforts were made to present a consistent and coherent theme throughout the design of BRANCH, many participants still expressed a desire for a greater level of consistency. However, it appeared from the views expressed in the focus groups that while participants unanimously agreed that a consistent and appealing design was integral to app usability, participants could not necessarily agree on the type of design which was the most appealing, with many different opinions being expressed. This identifies another usability barrier for BRANCH, ie, providing an aesthetic that is agreeable to all.

It looks a bit like it was maybe made on Word. So it just needs to be a bit more like corporate.

P3; Focus group 2

It was very green.

P4; Focus group 2

I didn’t find it that exciting and fun to go into like other apps...they’re colourful and, you know, didn’t find it that...found it quite bland.

P2; Focus group 2

Overall, this theme highlights how important a well-considered design is to the usability of an app. Participants reported that to promote usability and subsequent motivation to engage with the app, information must be clearly and succinctly provided, with standout features and a consistent design throughout.

#### Content

Maximized personalization of app content emerged as an important usability issue. Personalization was presented as a two-fold concept where participants wanted the app to be both automatically personalized to them as much as possible, but they also wanted the autonomy to personalize the app by themselves. Providing autonomy gave participants a sense of empowerment over their interactions with the app, and made them feel in control of their drinking.

While participants commonly enjoyed the personalization of the motivators and feedback features, there were aspects of the app that some reported could be improved. A particular issue was the daily newsfeed messages. These messages were sent to the entire user-base each day and were generic and not targeted to the individual. The majority said that these messages were not relatable (See Multimedia Appendix 4). Indeed, participants appeared to adeptly identify any area of the app that was not specifically tailored to each user. Surprisingly, this is in spite of the messages being written by the user-group of young adults with whom the app was developed. For the focus group participants, it seemed that personalization to every aspect of the individual, such as motivations for use, preferences for style and content, and even targeting geographical area, were usability enablers.

Equally, while participants wanted maximum automated personalization, they also wanted independence and autonomy to personalize the app by themselves. For example they wanted to be able to select whose posts were visible on their newsfeed and to be able to personalize the colors and content in the app. Having the option to personalize the app made them feel that the app was unique, belonging to them, and tailored specifically to their own preferences, which made the app more relatable and appeared to increase the usability of the app:

Cause if it was personalise...you could say that I don't like this kind of tip or I don't like certain things, and then you could kind of have it specific to you...

P3; Focus group 2

Or you [could] save it for later. ‘Cause a lot of these things...could be something you might not need now but in a month’s time you might think, oh, let me have a look on that app.

P7; Focus group 2

A novel feature of the app was the “social” component, where participants could post messages and interact with other users through the newsfeed and a “teams” page where users collected points for engaging with the app (see Multimedia Appendix 3). The majority of participants found the newsfeed feature a useful way to connect with other users, which made them feel like they were part of a community of like-minded others. Participants compared the newsfeed feature with other social media apps they enjoyed using, like Facebook, and reflected on how the newsfeed elevated using the app from an isolated, solitary activity to something that is shared with and connected to others. Participants also found it a useful tool to be able to compare their experiences with that of others, which enabled participants to normalize their experiences and motivated them to both continue using the app and to continue their drinking goals.

However, not all participants had a positive view on connecting with other users on the newsfeed. As the newsfeed connected users together who did not know each other, some participants reported that connecting with strangers was irrelevant to them. Participants held conflicting views on this issue and discussed how they had different ways of using online social tools, with no single model being suitable for everybody. Some users liked to be very active, while others did not want to be involved at all. In general, participants wanted the flexibility to be able to choose how involved they were with the social features:

You've got the danger of weirdoes and all that kind of stuff...

P7; Focus group 1

I mean I don't care what Steve from Birmingham has got...it's irrelevant for me...I would opt out of other people’s comments.

P3; Focus group 1

I liked hearing other people’s struggle and I liked hearing about other people’s triumphs. I didn’t feel so bad when I kind of, you know, fell off the wagon myself, okay, it’s not just me.

P5; Focus group 1

While the social feature of the newsfeed was highly praised by the participants, the teams section of the app was one of the most criticized areas. Participants reported that the teams concept was underdeveloped and not engaging to use. In particular, most of the participants mentioned that the objective of joining a team and the benefits of the feature, (which provided points for using the app) were not clear. However, participants generally still thought the feature had merit but that it needed to be improved. One method participants suggested to improve the usability of the Teams section, was to make it more socially interactive, and have a live feed where users could interact specifically with people in their team. However, as mentioned above, some participants were hesitant about interacting with people they did not know, and that interactions with friends would be more meaningful:

I think what needs to be really clear is once you’ve picked a team and once you’re in a team what can you do?

P5; Focus group 3

What do you get for winning? Otherwise it all just seems a bit pointless.

P3; Focus group 3

I’d just quite like to...go with my six or seven mates who all play football...and then your newsfeed is based around the team that you choose, so you see what your mates are saying.

P6; Focus group 3

Overall, the option for social connectivity within the app was a highly praised feature, which participants strongly believed improved the usability by fostering an engaging and interesting user experience that could be shared with others. However, participants also highlighted the need for improvement in the teams section, making the objectives and concepts clearer to understand and also increasing the level of social connectivity.

## Discussion

### Principal Findings

The aim of this study was to explore the experiences of enablers and barriers to usability for a prototype eSBI app called BRANCH targeting harmful drinking in young adults. The study found that an easy-to-use interface, with minimum required user-input and high levels of automated functioning were the most important usability requirements for participants. It also found that clear, consistent, and visually appealing design was integral to the level of usability. The option for social connectivity was important to participants, as were high levels of personalization. Poor functionality, text heavy content, high user-burden costs in time and effort, and unappealing design were considered major usability barriers. This study showed how focus group interviews can be used to get detailed feedback on the usability of an alcohol app, which can then be used to improve its effectiveness and ultimately increase its potential for reducing drinking among users.

The findings of this study are consistent with previous research, which found that difficulties in inputting data were among the most frequently criticized functionality issues in existing eSBI alcohol apps [3]. High data entry burden costs, which were considered a usability barrier by participants using BRANCH, have been reported to be a primary reason why people stop using health apps [27]. Participants wanted features that reduced the amount of time and effort required from them. They suggested that reminders, guided walkthroughs, and reduced scrolling were all features that could improve usability. The level of data participants are expected to enter into self-monitoring apps should be carefully considered in future app development [27]. Indeed, frustration with poor performance is one of the most common complaints of app users and results in apps being deleted entirely [28].

A well-conceived visual design was integral to the usability of BRANCH. Participants wanted clear, concise presentation of information which was not text heavy, a finding which is consistent with previous research [7]. Poor design, such as features and buttons not standing out, inhibit use as users are unable to distinguish between features. This has implications not only for usability but also for the potential effectiveness of the intervention. If an eSBI user cannot identify that a particular feature is available, then the user will not be exposed to the targeted alcohol harm reduction intervention. Furthermore, visual design influences the credibility of an app and users are more likely to rate consumer health information on the web as credible if it is presented in an aesthetic style [29].

An issue closely related to usability was engagement. Engagement refers both to how a user interacts with a technology and their emotional response to it [30-32]. For BRANCH, participants often stated how positive experiences of usability made them engage more with the app, making them more likely to keep on using it. For example, participants praised the newsfeed feature as one which enhanced their experience of app usability, as it provided them with meaningful interactions with other users and a sense of community. Participants also praised how personalization made the app feel more tailored to their own needs, providing a positive user experience. This is consistent with the elaboration likelihood model (ELM), which proposes that people are more motivated to engage with and process information more thoroughly if the message is personally relevant and meaningful [33]. The theoretical model of user engagement by Short et al [34] proposes that an individual’s characteristics and personal circumstances may influence their user experience of the app. It may be that future applications can enhance usability by targeting features and increasing personalization to target specific user characteristics.

Not improving usability would result in frustrating features and boredom is associated with disengagement with online programs [35]. The teams section in BRANCH, where participants were awarded points for engaging with the app, was criticized for being a major usability barrier because the objective of the feature came across as confusing to participants. While gamification methods (the use of gaming design in nongaming contexts) are popular and effective methods with which to improve engagement [36], it is apparent from the present study that the design of such features needs to be carefully considered in the context of the intervention, otherwise its relevance will be challenged.

Engagement is an ongoing issue for health app development. Issues such as low login rates and limited use of intervention features are consistently reported in literature [37,38]. Findings from the online intervention “Down Your Drink” reported that only 6% of users stayed with the program until the end of the 6 week program [39]. Enhancing the usability of engagement features is crucial to the effectiveness of an eSBI app. The more usable an app is, the more likely an individual is to revisit it and repeatedly use the program intervention features. Indeed, higher engagement through logins and repeated use is associated with better participant health outcomes [38,40,41].

### Conclusions

Optimizing usability for eSBI apps is a critical step in the development process. Consumer expectations for digital products are high and if products do not meet their expectations, then they may cease to use them. A recent survey demonstrated that peoples’ tolerance for poor performing apps has reduced, with approximately 50% of people reporting that they are less tolerant of problems in apps they use compared to a few years ago [28]. In case of young adults, the age group with the highest use of health apps [27], if an app does not function as they want it to, regardless of its objective, they will delete it. It is not good enough to have only a strong evidence-based core intervention, the whole package of delivery, including design, aesthetics, usability, and functionality needs to be iteratively refined and improved. As there are high demands on apps such as BRANCH that target skilled technology users such as young adults, the development of future eSBI apps that allows for usability testing with representative users may help support the effectiveness of eSBI to reduce harmful drinking. The BRANCH app is currently being evaluated as part of a randomized controlled trial with results expected in early 2018.

### Limitations

Significant efforts were made to recruit a sample of young adults, both male and female; however, the majority of participants (90%) were female. This is consistent with a previous study for a Web-based alcohol intervention, which also reported a high sample of females (73%) [42]. The advertisement was designed for both men and women, however more females replied to the advert and requested to bring along more female friends, creating a multiplying effect. While potentially introducing bias, there is research to suggest that women are more motivated to use the Internet for seeking health information [43,44] and are more likely to use eHealth interventions as recommended [45]. Therefore, the sample in the current study may represent the type of individuals more likely to engage with the Web-based BRANCH app. Future studies may wish to explore these differences in more detail, examining how males and females engage with eSBI, informing how interventions can be tailored to gender. Participants were not screened on their intention to cut down their alcohol use. Consequently, there may have been differences between the participants in the current study and some end-users in terms of how motivated they would be to engage with the app. However, BRANCH was not designed exclusively for those wishing to cut down, but also for those wanting to monitor their use or learn more about the risks of drinking. While this data was not collected, there were 20 participants in the present study who would have likely held a range of reasons for wanting to participate in the study, reflecting the target end-user of the app. Future studies should be further improved by specifically targeting at recruitment stage the types of end users the app is designed for. Focus groups can be subject to response bias, where participants provide answers based on what they think the researchers want to hear. However the findings in the current research reflect those in previous research both from the alcohol field [3,7] and from usability testing research in the computer science disciplines, suggesting that the results have meaning across different populations. Focus groups may also limit the full range of views due to convergence of ideas. Future research may wish to conduct both 1:1 as well as focus group interviews. The participants in the focus group scored high on the AUDIT. Alcohol is a topic seen as sensitive and stigmatizing, therefore participants may not have been comfortable sharing all of their experiences of using the app, as this may reveal details about their level of drinking.

## Appendix

Multimedia Appendix 1 BRANCH prototype app: drinking diary.

Multimedia Appendix 2 BRANCH prototype app: goals and information sections.

Multimedia Appendix 3 BRANCH prototype app: selecting a motivator, the Newsfeed, and Teams sections.

Multimedia Appendix 4 Automated messaging examples.

## Abbreviations

AUDIT: Alcohol use disorder identification test
ELM: Elaboration likelihood model
eSBI: Electronic screening and brief intervention
FRAMES: Feedback, Responsibility, Advice, Menu of options, Empathy, Self-efficacy
UCD: User-centered design
