# Supplementary figures and images for: Usability Testing of the BRANCH Smartphone App Designed to Reduce Harmful Drinking in Young Adults
Source: JMIR Mhealth Uhealth. 2017 Aug 8;5(8):e109. doi: 10.2196/mhealth.7836 (PMC5566629; doi:10.2196/mhealth.7836)

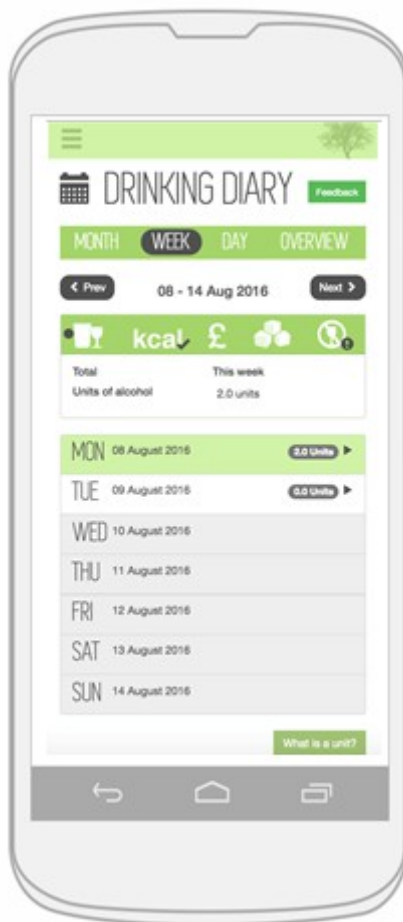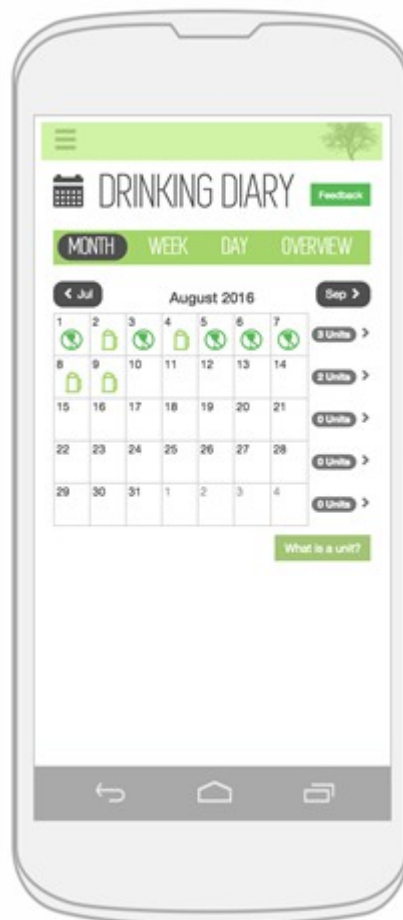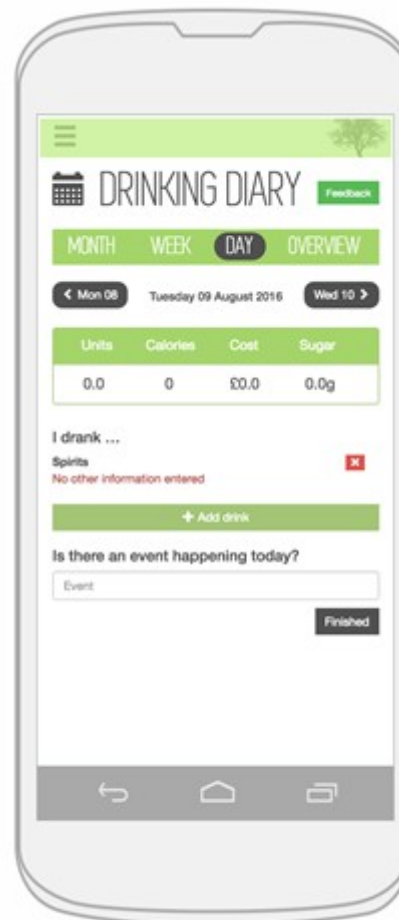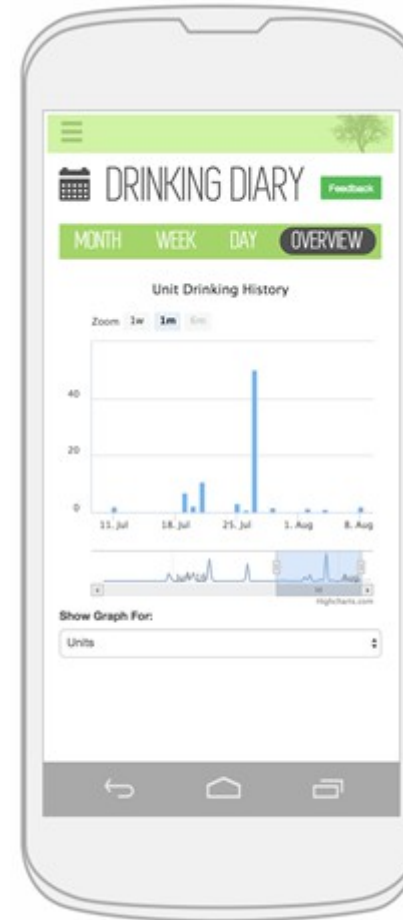

Supplement: Multimedia Appendix 1 [file mhealth_v5i8e109_app1.pdf]

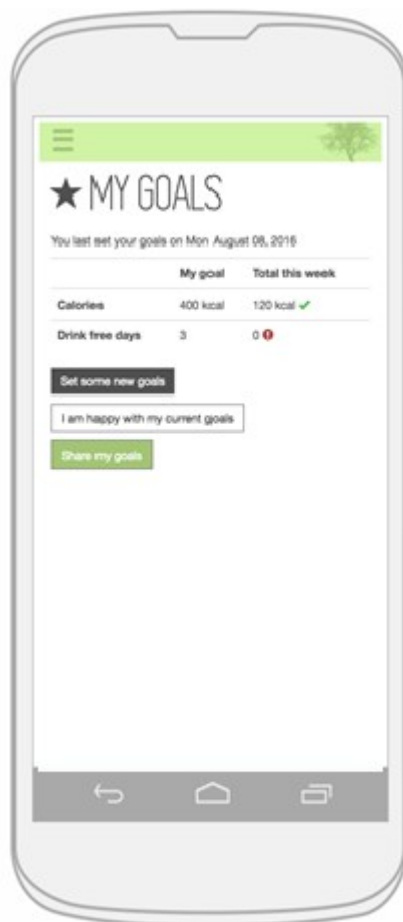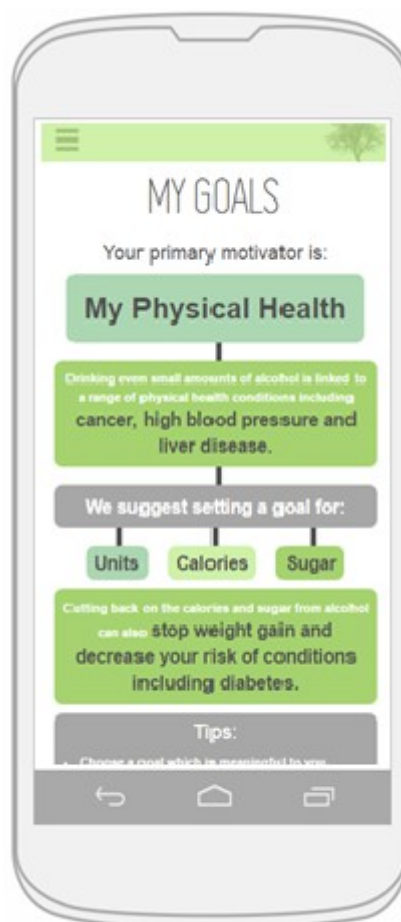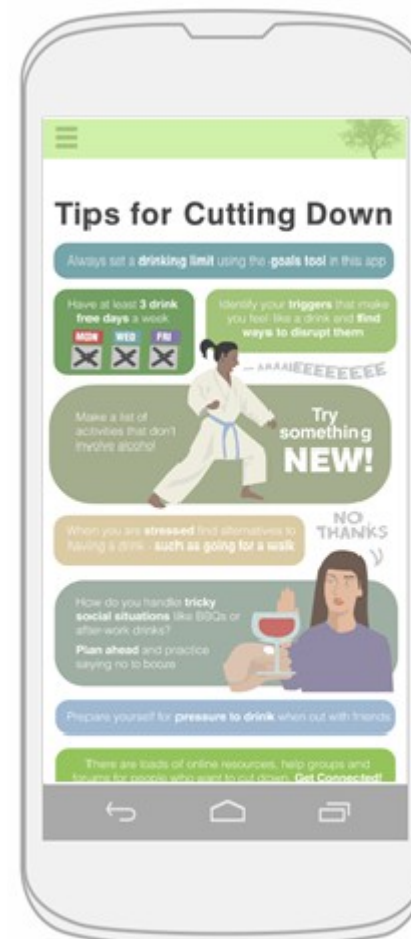

Supplement: Multimedia Appendix 2 [file mhealth_v5i8e109_app2.pdf]

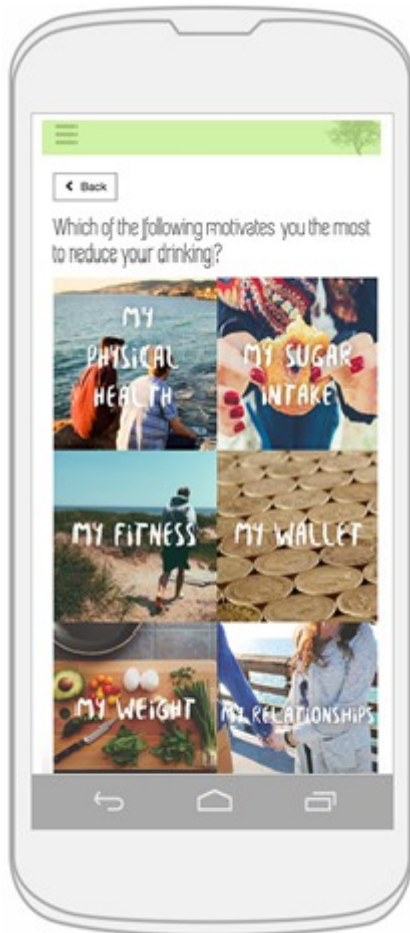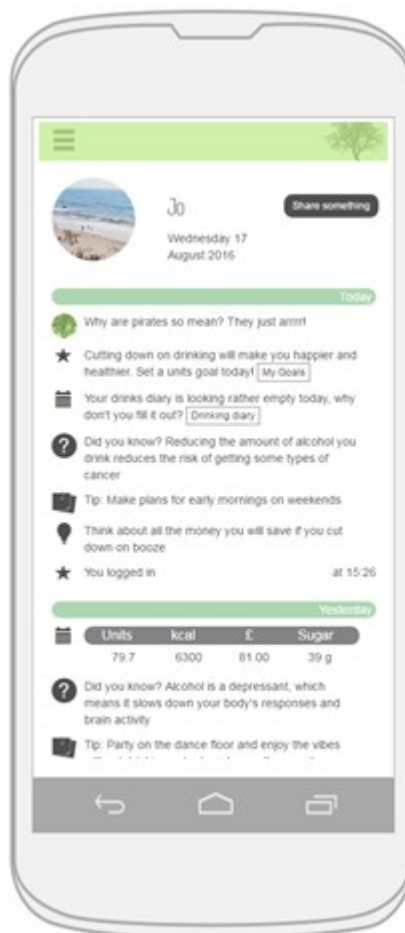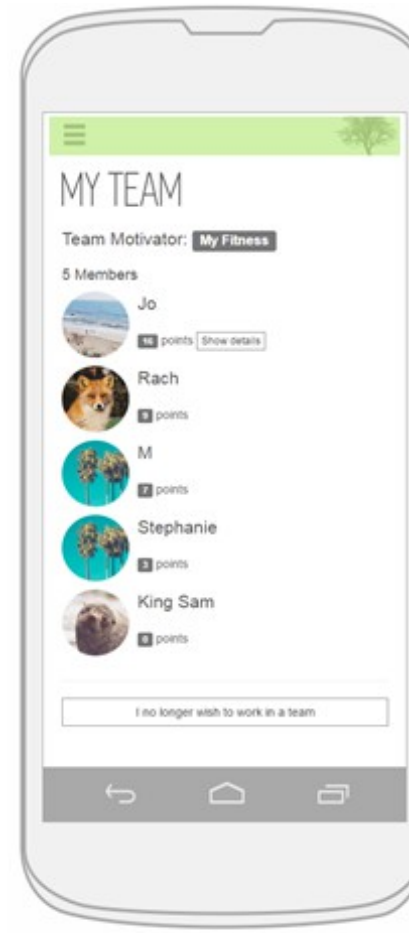

Supplement: Multimedia Appendix 3 [file mhealth_v5i8e109_app3.pdf]
