# Supplementary material for: Usability Testing of the BRANCH Smartphone App Designed to Reduce Harmful Drinking in Young Adults
Source: JMIR Mhealth Uhealth. 2017 Aug 8;5(8):e109. doi: 10.2196/mhealth.7836 (PMC5566629; doi:10.2196/mhealth.7836)
Supplement: Multimedia Appendix 4 [file mhealth_v5i8e109_app4.pdf]

Type of message	Message
Encouragement to set a goal	Start to take control of how much you are spending on alcohol: Set a cost goal today
Praise when goal is set	Well done for setting a cost goal - you're in control now!
Motivation during goal	Remember your cost goal. Don't lose momentum now!
End of goal message (success)	Congratulations on reaching your costs goal - why don't you set your next goal now?
End of goal message (failure)	Oh dear, it looks like you didn't achieve your cost goal. Don't despair, set another goal now, you'll get it next time!
Motivational message (general)	Do you think your drinking is a positive part of your lifestyle?
Did you know?	We all have tough moments, keep at it!
Tip	Make plans for early mornings on weekends
